# Editorial on the Paper “The Epidemiology and Genetics of Hyperuricemia and Gout across Major Racial Groups: A Literature Review and Population Genetics Secondary Database Analysis” by Butler, Alghoubayshi and Roman

**DOI:** 10.3390/jpm12081331

**Published:** 2022-08-19

**Authors:** Farah R. Zahir

**Affiliations:** 1 Irfa’a Foundation, Burlington, ON L7L 5H6, Canada; farahz@bcchr.ca; 2 Department of Medical Genetics, University of British Columbia, Vancouver, BC V6H 3N1, Canada

This report by Butler et al. is well conceived, executed and will be a useful addition to the literature on Hyperuricemia (HU) and gout. Gout, estimated to affect ~4% of the population [1], is a common condition. However, while there has been an exponential increase in the number of publications on this disorder over the past decade (a quick PubMed search using the word “gout” suffices to prove the point), the complete causative and epidemiological profile has not yet been clearly established. While there are a growing number of studies probing the epidemiology for the condition [2] this report is significant in that it aims to stratify a population-based risk allele profile. The authors compare the genotypes for 11 SNPs that have been reported in the literature to be associated with or causative for HU/gout, across four sub-populations; Africans in Southwest U.S. (ASW), Han-Chinese (CHS), Japanese (JPT), and Mexicans (MXL) and to the major population, Europeans (EUR). They accomplish this by accessing population specific genotypes for the SNPs from the 1000 genomes project. This is an important consideration, and the authors are to be commended for looking at the population-specific stratification of risk. Indeed, their finding of a significant increase in the prevalence of the risk allele, sometimes for all 11 SNPS in certain ethnic populations, corroborates the importance of the notion. However, the very low number of individuals in the test sample groups—on average 100–200 individuals per sub-population—is a concern. It is also somewhat concerning that the predominant EUR sample group has about ten times the number of individuals, at ~1000. This raises the question whether the authors assumed that all individuals in the 1000 genomes project were “EUR” or whether it so happened that there were that many more EUR subjects than the other ethnic groups in the 1000 genomes database. Nevertheless, appropriate statistical corrections were applied, and the trend of findings is in keeping with other reports which also note significant ethnicity-based risk [3].

The authors frankly discuss the limitations of their work, as well as include a section on the variety of populations in “Asia” which makes generalizing about “one Asian” population difficult. This point was particularly refreshing, as unfortunately too many researchers lump together all of the extremely genetically diverse region that is Asia (for example, Malaysia alone has over 50 ethnic groups) into one ethnicity. Among the discussed limitations is the possibility that other risk alleles, not included in this study, may exist. Despite the limitations, this is a step in the right direction, and given the thousands of publicly available racially filterable genomes/exomes currently, it would be interesting to see the study reproduced with much larger sample numbers.

In summary, works such as this, which attempt to probe the distribution of risk alleles among ethnic groups, are vitally important in our world. Even though at least up until recently, genetic studies have predominantly focused on the Caucasian populations, the scientific community is now catching up to the fact that we need to understand disease genetics from the point of view of ethnic genetic background, in order to achieve our precision medicine goals. Papers such as that of Butler et al. are a step in the right direction and add further proof to the need to carry out more work on this subject.

## References

[1] Chen-Xu M., Yokose C., Rai S.K., Pillinger M.H., Choi H.K. (2019). Contemporary prevalence of gout and hyperuricemia in the United States and decadal trends: The National Health and Nutrition Examination Survey, 2007–2016. Arthritis Rheumatol..

[2] Singh J.A., Gaffo A. (2020). Gout epidemiology and comorbidities. Semin Arthritis Rheum..

[3] Kuo C.F., Grainge M.J., Zhang W., Doherty M. (2015). Global epidemiology of gout: Prevalence, incidence and risk factors. Nat. Rev. Rheumatol..

